# Supplementary material for: Aza-BODIPY: A New Vector for Enhanced Theranostic Boron Neutron Capture Therapy Applications
Source: Cells. 2020 Aug 25;9(9):1953. doi: 10.3390/cells9091953 (PMC7565158; doi:10.3390/cells9091953)
Supplement: Supplementary file 1 [file cells-09-01953-s001.pdf]

cells

Supplementary information

Theranostic aza-BODIPY as vector for enhanced Boron Neutron Capture Therapy applications

Ghadir Kalot [1,†], Amélie Godard [2,†], Benoît Busser [1,3], Jacques Pliquett [2], Mans Broekgaarden [1], Vincent Motto-Ros [4], K. David Wegner [5], Ute Resch-Genger [5], Ulli Köster [6], Franck Denat [2], Jean-Luc Coll [1], Ewen Bodio [2*], Christine Goze [2,*] and Lucie Sancey [1,*]

Materials and methods for chemical synthesis and characterization

Reactions were carried out in analytical reagent grade solvents from Carlo Erba under normal atmosphere. Dry solvents, purchased from Carlo Erba, were non-stabilized and dried using a MB-SPS-800 (MBraun) or PureSolv-MD-5 (Inert®). All reagents purchased from Sigma Aldrich™, Thermo Fisher Scientific™ or ACROS Organics™ were used as received without further purification. Sodium mercaptododecaborate (^{10}B) was purchased from Katchem™. Reactions were monitored by thin-layer chromatography and RP-HPLC-MS. Analytical thin-layer chromatography was performed with Merck 60 F254 silica gel (precoated sheets, 0.2 mm thick). Column chromatography was carried out using silica gel (Sigma Aldrich; 40-63 µm 230-400 mesh 60Å). Ion exchange was executed using an Amberlite™ IRA410Cl ion-exchange resin.

(^{1}H, ^{11}B, ^{13}C, ^{19}F)-NMR spectra were recorded at 298 or 343 K on Bruker 500 Avance III or 600 Avance III spectrometers. Chemical shifts are given relative to TMS (^{1}H, ^{13}C), BF_3*Et_2O (^{11}B, ^{10}B), $CFCl_3$ (^{19}F), and were referenced to the residual solvent signal. High resolution mass spectra (HR-MS) were recorded on a Thermo LTQ Orbitrap XL ESIMS spectrometer. NMR and mass-analyses were performed at the "Plateforme d'Analyse Chimique et de Synthèse Moléculaire de l'Université de Bourgogne" (PACSMUB).

HPLC-MS analyses were obtained from a Thermo-Dionex Ultimate 3000 instrument (pump + autosampler at 20 °C + column oven at 25 °C) equipped with a diode array detector (Thermo-Dionex DAD 3000-RS) and a MSQ Plus single quadrupole mass spectrometer equipped with Phenomenex Kinetex® column (2.6 µm C18 100 Å, LC Column 50 x 2.1 mm).

The employed gradient for analyses was as follows:

Time [min]	% H_2O + 0.1% formic acid	% ACN + 0.1% formic acid	Flow [mL/min]
**0**	95	5	0.5
**5**	0	100	0.5
**6.5**	0	100	0.5
**6.6**	95	5	0.5
**8.5**	95	5	0.5
**8.51**	95	5	0.05

Table S1: HPLC analytical gradient.

Semi-preparative separations were executed on a HPLC-system, from Shimadzu, that was equipped with 2 LC-20AT pumps, a SPD-20A UV/Vis detector, a FRC-10A fraction collector, a SIL-10AP sampler and a CBM-20A control unit. The column was a Shim-Pack GIST 5 µm C18 10x250 mm column obtained from Shimadzu too. The gradient using a mixture of ACN and water with 0.1% TFA and a flow rate of 5 mL/min was as follows:

Time [min]	% H_2O + 0.1 %TFA	% ACN + 0.1 %TFA	Flow [mL/min]
**0**	75	25	5
**5**	75	25	5
**25**	0	100	5
**28**	0	100	5
**30**	75	25	5

Table S2: Detailed of gradients.

Synthesis and characterization

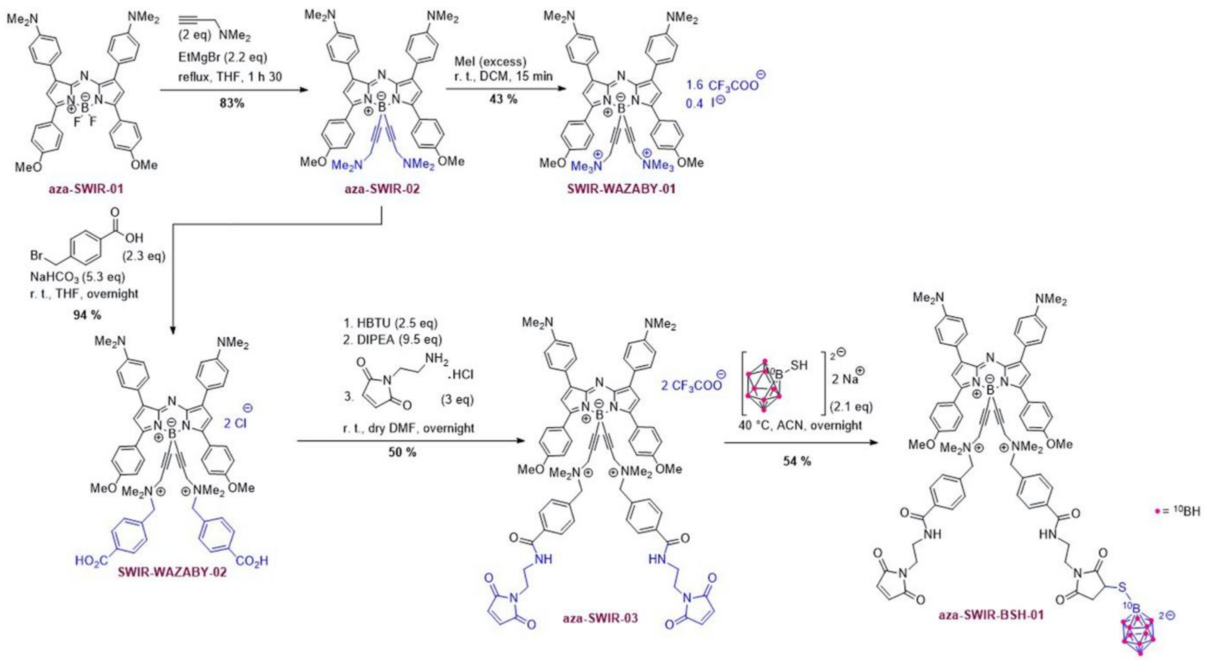

Scheme S1: Synthetic pathways of **aza-SWIR-BSH-01**.

Aza-SWIR-01, **aza-SWIR-02**, and **SWIR-WAZABY-01** were synthesized according to procedures, we previously reported [1].

- Compound **SWIR-WAZABY-02**:

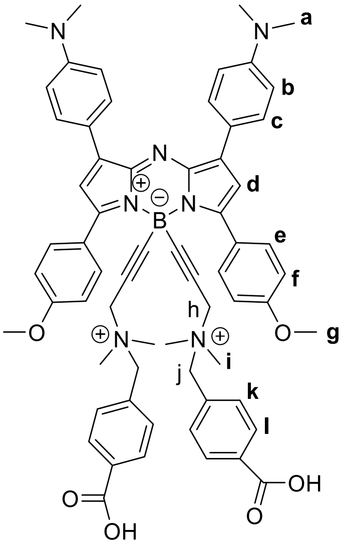

$C_{64}H_{66}BN_7O_6^{2+} + 2Cl^-$
M = 1110.99 g.mol^{-1}

SWIR-WAZABY-02

Aza-SWIR-02 (250 mg, 0.3 mmol, 1 eq) was added in a 100 mL round bottom flask and dissolved in a mixture of THF and water (50 mL / 8 mL). $NaHCO_3$ (137 mg; 1.6 mmol; 5.3 eq) and 4-bromomethylbenzoic acid (144 mg; 0.7 mmol; 2.3 eq) were added successively. The resulting solution was stirred at room temperature (RT) overnight and transferred to a separating funnel. Then, 90 mL of Et_2O and 90 mL of water were added and the two layers were separated. The aqueous layer was washed with Et_2O (6 × 60 mL) to remove the remaining traces of 4-bromomethylbenzoic acid. Then, the aqueous layer was evaporated at 35 °C to 2/3th of the volume and 10 mL of HCl_{aq} (3M) were added to precipitate the compound (via protonation of the carboxylate groups). The mixture was centrifuged during 5 min at 6 000 RPM. The supernatant was removed and the sediment was suspended in Et_2O (15 mL) and centrifuged. The process was repeated 3 times and the solid was dried under vacuum to give pure **SWIR-WAZABY-02** as a crystalline dark powder (336 mg; 0.3 mmol; 94% yield). ^{1}H NMR (500 MHz, 298 K, MeOD-d$_4$) δ (ppm) = 2.88 (s, 12H), 3.35 (s, 12H), 3.83 (s, 6H), 3.98 (s, 4H), 4.28 (s, 4H), 7.16 (d, *J* = 9.0 Hz, 4H), 7.44 (d, *J* = 8.2 Hz, 4H), 7.55 (s, 2H), 7.88 (d, *J* = 9.0, 4H), 7.98 (d, *J* = 8.2 Hz, 4H), 8.33 (d, *J* = 9.0 Hz, 4H), 8.46 (d, *J* = 9.0 Hz, 4H).^{13}C NMR (125 MHz, 298 K, MeOD-d$_4$) δ (ppm) = 48.0, 50.7, 52.1, 56.7, 57.5, 67.8, 89.6, 116.5, 123.1, 123.3, 125.8, 131.9, 132.2, 133.1, 133.6, 134.9, 135.2, 135.7, 142.3, 145.1, 145.6, 160.2, 164.9, 169.2. ^{11}B NMR (193 MHz, 298K, MeOD-d$_4$) δ (ppm) = -11.63 (bs). HR-MS (ESI): m/z calculated for $^{12}C_{64}{}^{1}H_{66}{}^{11}B^{14}N_7{}^{16}O_6^{2+}$ [M]$^{2+}$ 519.75783 Th; found 519.75905 Th. Analytical HPLC: Tr = 4.50 min.

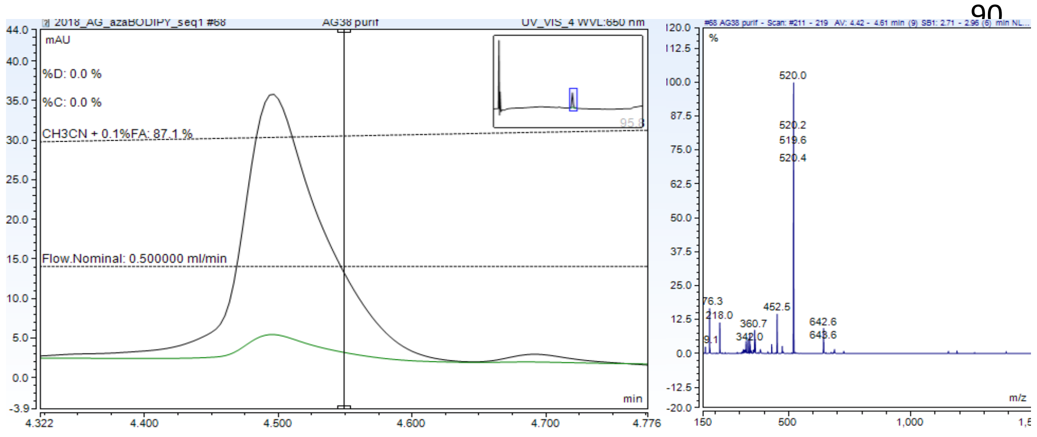

Figure S1: Analytical HPLC of **SWIR-WAZABY-02**

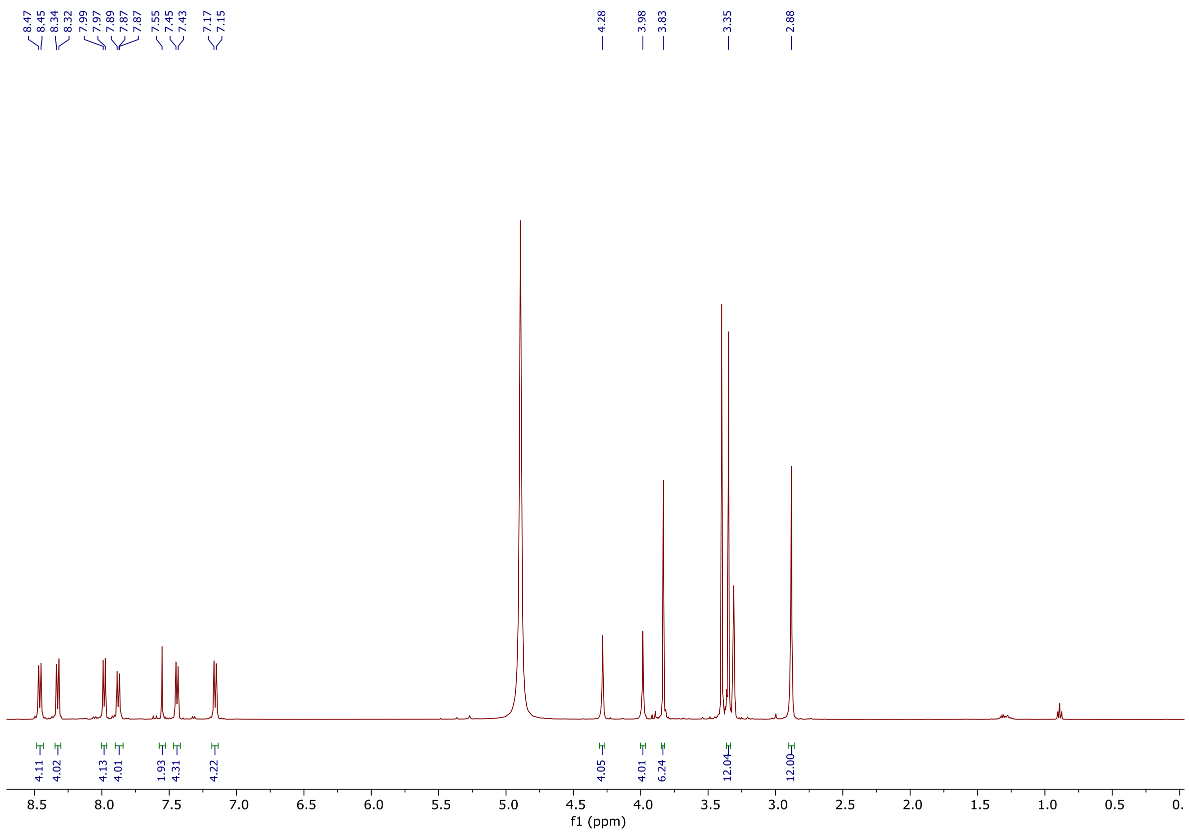

Figure S2: ^{1}H NMR of **SWIR-WAZABY-02** (500 MHz, 298 K, MeOD-d$_4$)

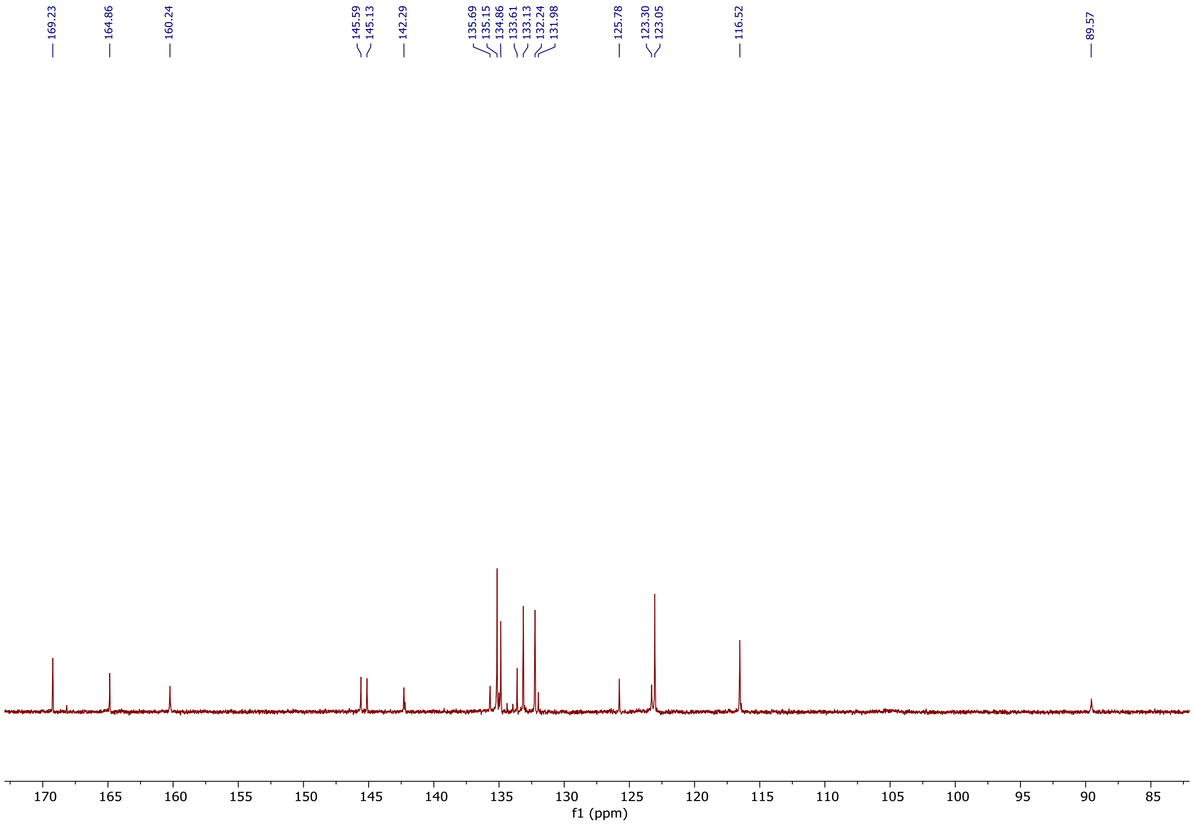

Figure S3: ^{13}C NMR of **SWIR-WAZABY-02** (125 MHz, 298 K, MeOD-d_4)

- Compound **aza-SWIR-03**:

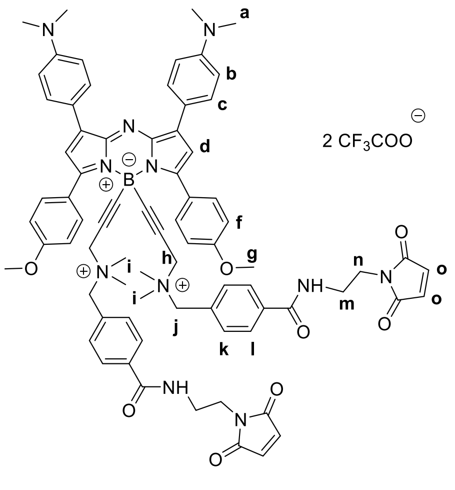

aza-SWIR-03

$C_{76}H_{78}BN_{11}O_8^{2+}$ + $2C_2F_3O_6^-$
M = 1510.37 g.mol^{-1}

SWIR-WAZABY-02 (250 mg, 0.2 mmol, 1 eq) was dissolved in 10 mL of dry DMF. HBTU (208 mg; 0.5 mmol; 2.5 eq) solubilized in dry DMF (10 mL) was added to the reaction medium. DIPEA (340 µL; 1.9 mmol; 9.5 eq) was then added and the reaction mixture was stirred at RT for 1 h. *N*-(2-aminoethyl)maleimide hydrochloride (0.6 mg, 0.6 mmol, 3 eq) was solubilized in dry DMF (10 mL) and added to the medium. The reaction mixture was stirred at RT overnight. The solvent was removed at 45°C under vacuum. The crude was purified by semi-preparative HPLC to obtain **aza-SWIR-03** as a green solid (151 mg, 50 % yield). ^1H NMR (500 MHz, 298 K, DMSO-d_6) δ = 2.74 (s, 12H), 3.07 (s, 12H), 3.40-3.43 (m, 8H), 3.73 (s, 6H), 3.92 (s, 4H), 4.16 (s, 4H), 6.86 (d, *J* = 9.0 Hz, 4H), 7.00 (s, 4H), 7.06 (d, J = 9.0 Hz, 4H), 7.27 (s, 2H), 7.37 (d, J = 8.0 Hz, 4H), 7.78 (d, *J* = 8.0 Hz, 4H), 8.11 (d, *J* = 9.0 Hz, 4H), 8.30 (d, *J* = 9.0 Hz, 4H), 8.65 (t, *J* = 5.9 Hz, 2H). ^{13}C NMR (150 MHz, 298 K, DMSO-d_6) *δ* (ppm) = 37.1, 37.8, 49.1, 54.0, 55.4, 64.7, 86.7, 112.1, 112.8, 113.8, 114.7, 116.0, 116.7, 118.6, 119.8, 124.3, 127.6, 130.1, 130.5, 132.0, 132.5, 134.6, 136.0, 141.6 , 142.1, 151.0, 155.9, 157.9, 158.1, 158.3, 158.6, 160.9, 165.6, 171.1. HR-MS (ESI): m/z calculated for $^{12}C_{76}{}^{1}H_{78}{}^{11}B^{14}N_{11}{}^{16}O_8^{2+}$ [M]$^{2+}$ 641.80585 Th; found 641.80752 Th. Analytical HPLC: Tr = 4.56 min.

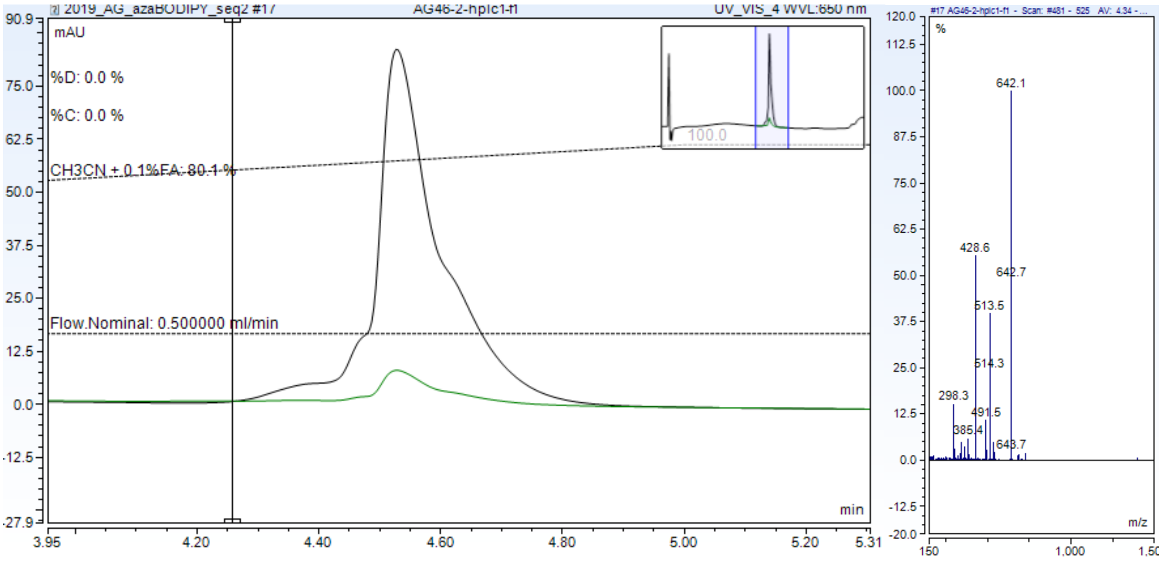

Figure S4: Analytical HPLC of **aza-SWIR-03**

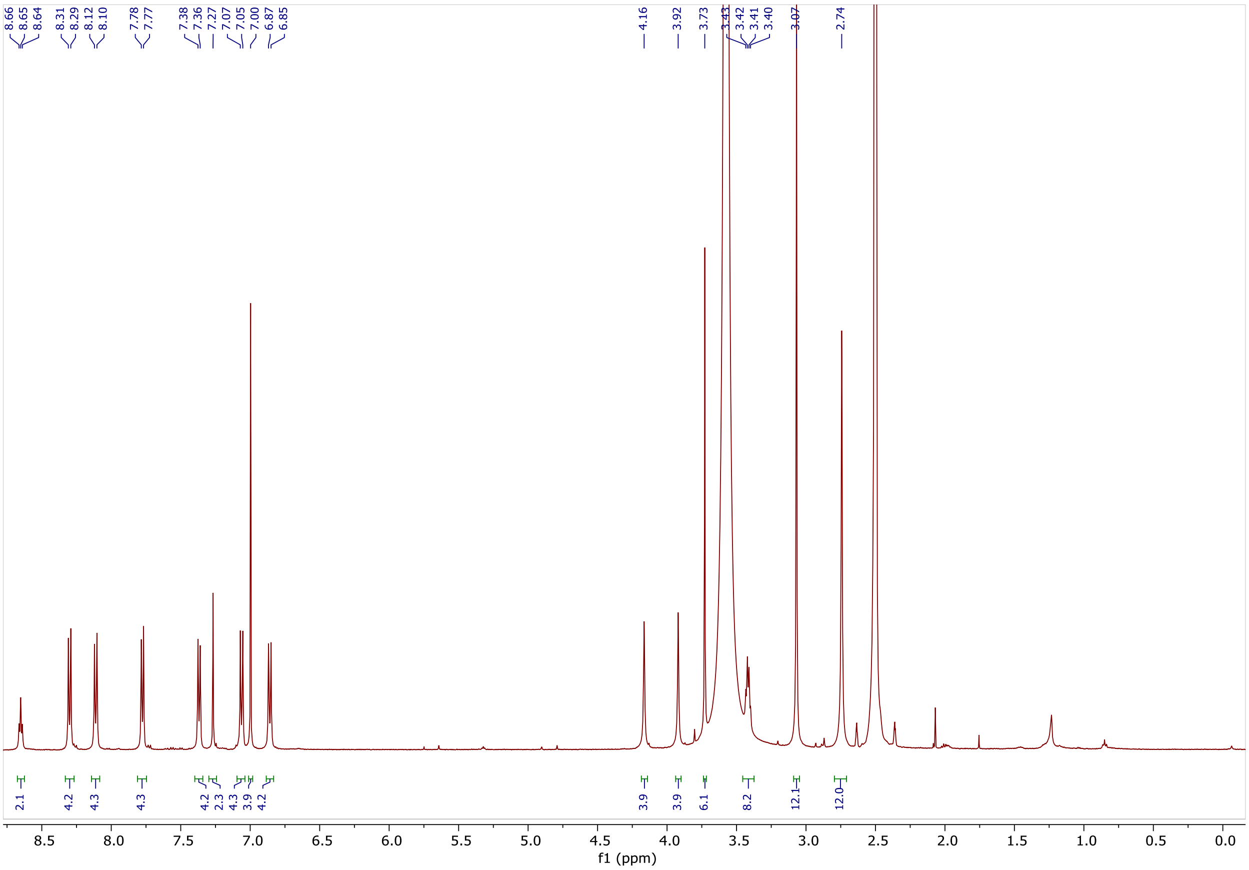

Figure S5: ^{1}H NMR of **aza-SWIR-03** (500 MHz, 298 K, DMSO-d_6)

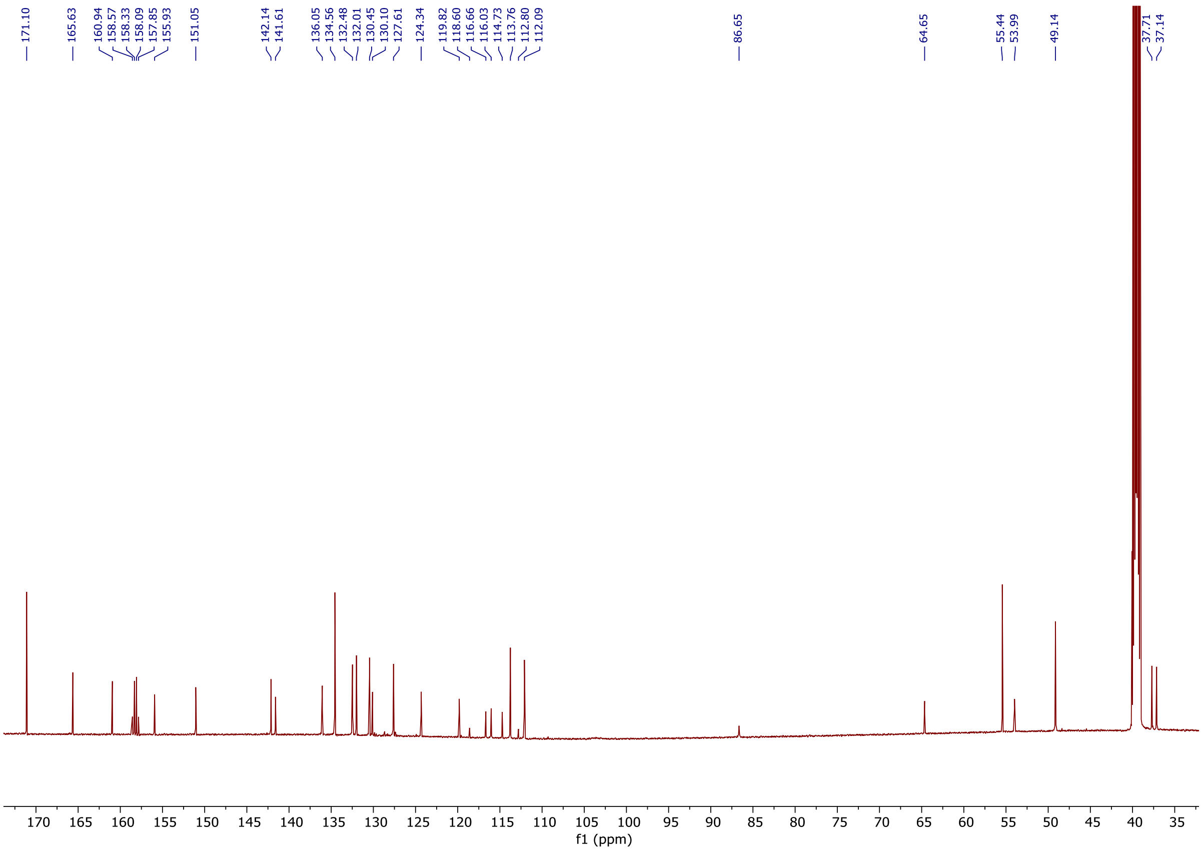

Figure S6: ^{13}C NMR of **aza-SWIR-03** (150 MHz, 298 K, DMSO-d6)

- Compound **aza-SWIR-BSH-01**:

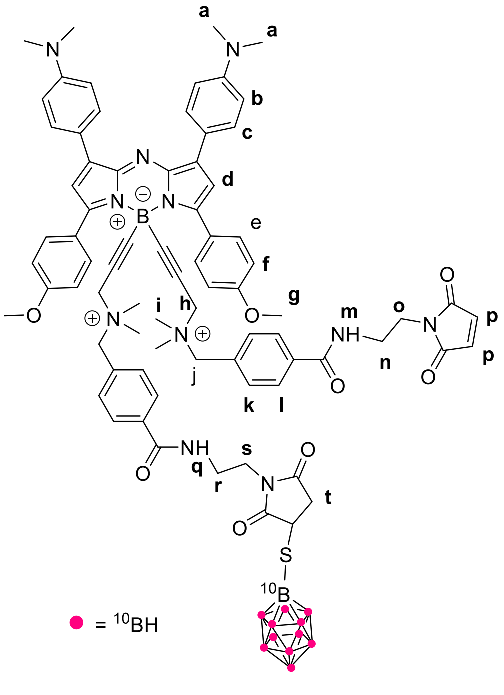

Aza-SWIR-BSH-01

$C_{76}H_{90}{}^{10}B_{12}N_{11}O_8S$
M = 1448.65 g.mol^{-1}

Aza-SWIR-03 (120 mg, 79 µmol, 1 eq) was dissolved in 4 mL of ACN in a 10 mL round-bottom flask. Sodium mercaptoundecahydrododecaborate (^{10}B) (42 mg; 166 µmol; 2.1 eq) was added and the reaction mixture was stirred overnight at 40 °C, leading to the formation of a precipitate. The mixture was centrifuged during 5 min at 6 000 RPM. The supernatant was removed and the sediment was suspended in ACN (15 mL), the process was repeated three times with ACN, once with DCM (15 mL), three time with Et_2O (15 mL) and twice with pentane (15 mL) to obtain **aza-SWIR-BSH-01** as a green solid (62 mg, 54%). ^1H NMR* (500 MHz, 298 K, DMSO-d_6) δ = 1.03 (bs, 11H), 2.73–2.78 (m, 14H), 3.01 (dd, *J* = 8.3; 18.4 Hz, 2H), 3.07 (s, 12H), 3.46–3.56 (m, 4H), 3.58 (t, *J* = 5.8 Hz, 2H), 3.62 (dd, *J* = 8.3; 3.1 Hz, 1H), 3.73–3.74 (m, 6H), 3.90–3.97 (m, 2H), 4.17 (t, *J* = 17.9 Hz, 4H), 6.85–6.89 (m, 4H), 6.99–7.00 (m, 2H), 7.07–7.09 (m, 4H), 7.26–7.28 (m, 2H), 7.36–7.39 (m, 4H), 7.76–7.82 (m, 4H), 8.10–8.13 (m, 4H), 8.30–8.33 (m, 4H), 8.43 (t, *J* = 5.8 Hz, 1H), 8.64 (t, *J* = 5.8 Hz, 1H). ^{13}C NMR (150 MHz, 343 K, DMSO-d_6) δ (ppm) = 36.9, 37.0, 37.1, 37.5, 40.7, 40.8, 41.9, 41.9, 49.1, 49.1, 54.2, 54.3, 55.4, 55.4, 64.9, 64.9, 65.0, 86.2, 111.9, 111.9, 112.0, 113.6, 113.6, 113.6, 115.8, 115.8, 115.8, 119.7, 119.9, 124.1, 124.2, 124.2, 127.4, 127.5, 127.5, 129.5, 129.6, 129.7, 129.8, 130.2, 130.2, 131.7, 131.7, 132.1, 132.1, 132.2, 132.2, 134.2, 136.1, 136.2, 136.3, 141.5, 142.0, 150.9, 151.0, 155.9, 156.0, 160.8, 160.8, 160.8, 165.5, 165.5, 165.6, 165.6, 170.7, 176.9, 178.5, 178.5. ^{11}B NMR (193 MHz, 343 K, DMSO-d_6) δ (ppm) = - 6.92 (bs), - 12.37 (bs), - 14.49 (s), - 15.44 (s), - 17.25 (bs). ^{10}B NMR (64 MHz, 343 K, DMSO-d_6) δ (ppm) = - 6.92 (bs), - 14.61 (s), - 15.52 (s). HR-MS (ESI): *m/z* calculated for $^{12}C_{76}{}^{1}H_{90}{}^{11}B^{10}B_{12}{}^{14}N_{11}{}^{16}O_8{}^{32}S^{23}Na_2{}^{2+}$ [M+2Na]$^{2+}$ 746.90622 Th; found 746.90811 Th. Analytical HPLC: Tr = 5.32 min. NMR spectra seems to indicate the presence of some amounts of another product, while there is no sign of it on HPLC chromatogram.

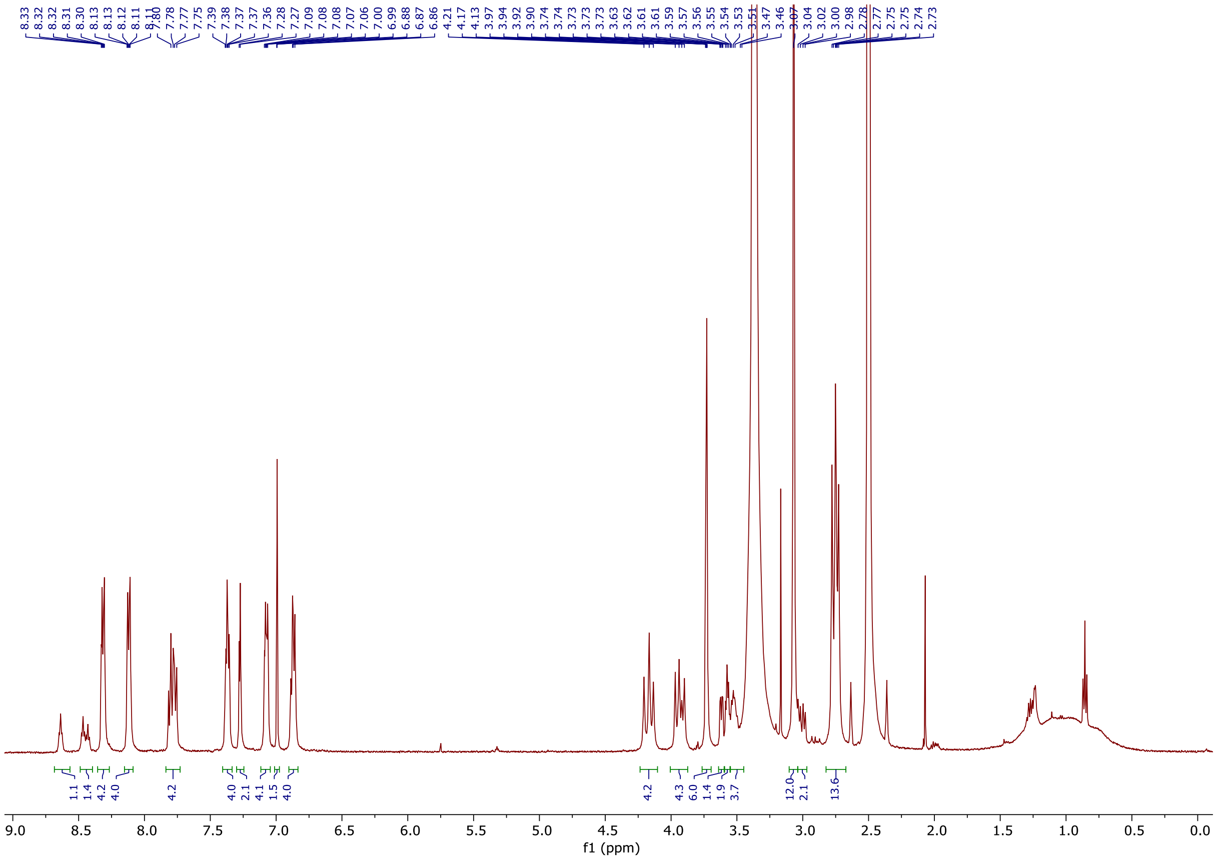

Figure S7: ^1H NMR of **aza-SWIR-BSH-01** (500 MHz, 298 K, DMSO-d_6).

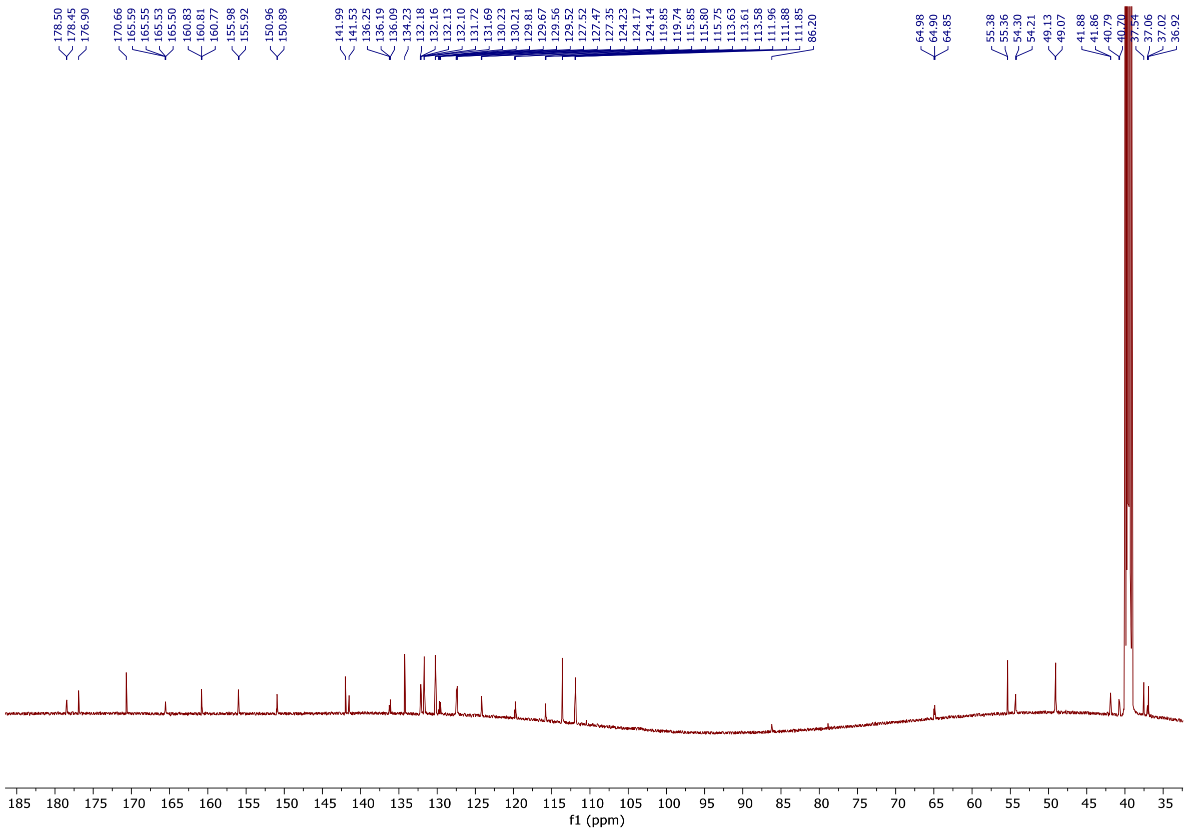

Figure S8: ^{13}C NMR of **aza-SWIR-BSH-01** (150 MHz, 343 K, DMSO-d$_6$.

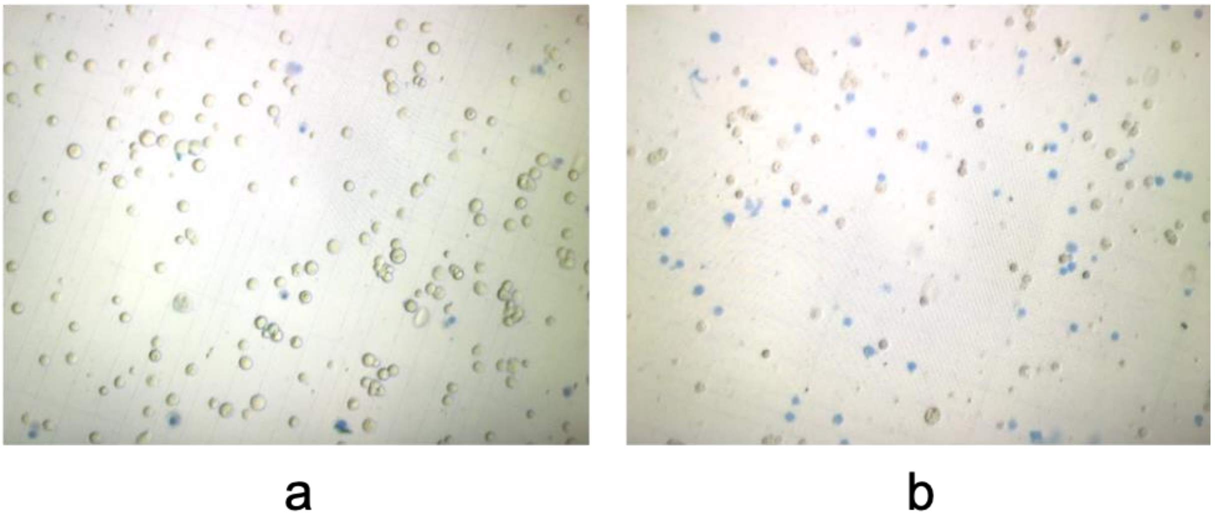

Figure S9: U-251 cells incubated with **aza-SWIR-BSH-01** before (a) and after (b) 10 min of neutron exposure. The clichés were taken from microscopy observation. Cells were diluted with Trypan blue to count the viable cells and exclude the dead cells (in blue) on Malassez slides. The presence of huge number of blue cells in (b) evidences the strong and immediate impact of the neutron exposure on cells incubated with **aza-SWIR-BSH-01**.

Reference

1. Godard, A.; Kalot, G.; Pliquett, J.; Busser, B.; Le Guevel, X.; Wegner, K.D.; Resch-Genger, U.; Rousselin, Y.; Coll, J.L.; Denat, F., et al. Water-Soluble Aza-BODIPYs: Biocompatible Organic Dyes for High Contrast In Vivo NIR-II Imaging. *Bioconjug. Chem.* **2020**, *31*, 1088-1092, doi:10.1021/acs.bioconjchem.0c00175.
